# Pharmacology of reflex blinks in the rat: a novel model for headache research

**DOI:** 10.1186/s10194-016-0686-x

**Published:** 2016-10-21

**Authors:** M. G. Jones, A. P. Andreou, S. B. McMahon, D. Spanswick

**Affiliations:** 1 Neurorestoration Group, Wolfson Centre for Age-Related Disease, Kings College London, London, UK; 2 Academic Headache Centre, Wolfson Centre for Age-Related Disease, Kings College London, London, UK; 3 London and Pain Management and Neuromodulation Centre, St Thomas’s Hospital, London, UK; 4 Neurosolutions Ltd., University of Warwick, Coventry, UK; 5 Zenith NeuroTech, Wolfson Centre for Age-Related Disease, Kings College London, London, UK

**Keywords:** Migraine, Blink reflex, Nitric oxide donor, Sumatriptan

## Abstract

**Background:**

Migraineurs are highly sensitive to the nitric oxide donor glyceryl trinitrate which triggers attacks in many sufferers. In animal studies, glyceryl trinitrate increases neuronal activity in the trigeminovascular pathway and elevates neurotransmitter levels in the brainstem. Many migraineurs also display alterations in blink reflexes, known to involve brainstem circuits. We investigated the effect of GTN on evoked blinks in the anaesthetised rat to determine whether such reflexes may prove useful as the basis for a novel animal model to evaluate potential anti-migraine therapeutic agents.

**Method:**

In anaesthetised rats the electromyogram associated with the reflex blink evoked by corneal airpuff was recorded. Rats were infused with glyceryl trinitrate, sumatriptan plus glyceryl trinitrate or vehicle control. Changes in the magnitude of the reflex blink-associated electromyogram following these treatments were measured.

**Results:**

Glyceryl trinitrate potentiated the evoked reflex blink-associated EMG response from 2 h after infusion. That effect was abolished by simultaneous infusion of sumatriptan with glyceryl trinitrate.

**Conclusions:**

These results show that simple skin surface measurements of evoked electromyographic activity in the rat can reliably detect the evoked blink reflex that can be potentiated by nitric oxide donors. This novel model may be an effective tool for evaluating putative anti-migraine therapeutic agents.

## Background

Migraine is a common and complex syndrome of the nervous system [21]. Although its pathogenesis remains unknown, it probably involves a cascade of events that include both the central and the peripheral nervous system. The trigeminal system appears to play an important role in the manifestation of head pain and trigeminovascular activation [22]. Centrally, the involvement of diencephalic and brainstem mechanisms that modulate the ascending trigeminal pathway is proposed by a number of clinical and pre-clinical studies to play, at least partially, some role during migraine attacks [1, 2, 4].

Modelling the activation of the trigeminal system and central brainstem alterations in animal models, has so far involved mainly invasive procedures and either single neuron electrophysiology or immunolabelling techniques [7]. Glyceryl trinitrate (GTN) infusion, which in migraineurs can induce a headache developing over the course of hours into full migraine [12, 42], has been also employed as a migraine model [8, 33]. In animal studies, using invasive procedures, GTN has been shown to increase neuronal activity of the trigeminovascular pathway and elevate neurotransmitter levels in the brainstem [20, 36, 38–40]. Peripherally, GTN has been shown to influence the expression of calcitonin gene-related peptide (CGRP) in the trigeminal ganglia, and more recently, to reveal a number of transcriptional changes [24]. Peripheral investigations in this model also included the actions of triptans on nitric oxide-induced dural vasodilatation [5]. Non-invasively, the GTN model has been used to study behavioral endpoints with potential clinical relevance in migraine, such as photophobia, facial expressions of pain and limb allodynia [11, 23]. However, these endpoints can be subjective and multiple GTN infusions may be required to reach a statistically significant change.

In humans a subtle method for recording activation of the trigeminal pathway and of brainstem mechanisms, is that of the blink reflex. The blink reflex has been used by a number of studies in humans to study brainstem connectivity with the trigeminal system [19, 29, 44]. Although the actual measurements are of an electromyographic activity, the blink reflex is an objective neurophysiological method that evaluates the connectivity of the lateral medulla and pons with the trigeminal and facial nerves. It involves stimulation of the ipsilateral supraorbital branch of the trigeminal nerve which results in an afferent response along the trigeminal nerve to the trigeminal nucleus in the pons and the nucleus of the spinal tract of the trigeminal nerve in the brainstem. Through a series of interneurons in the pons and lateral medulla, the nerve impulse next reaches the ipsilateral and contralateral facial nuclei, from which the efferent signal travels along the facial nerve bilaterally. The resultant electropmyographic activity will have an R1 and a delayed R2 component representing these oligo- and poly-synaptic pathways in the brainstem between the sensory trigeminal nucleus and the motor facial nucleus [13, 18]. In migraine patients, a number of studies suggested altered responses during and outside a migraine attack [9, 15, 46], although the affected components differ in some of these reports. Increased blink reflex responses in the interictal phase and during migraine attacks have been recorded [27, 28]. Studies investigating the habituation phenomenon have shown reduced blink reflex habituation responses during the premonitory stages of migraine attacks [14, 16], that in the majority of patients, normalise during a migraine attack [27]. More interestingly, GTN infusion in healthy volunteers is shown to induce comparable changes in nociceptive blink reflex, to those found immediately before and during an attack in migraineurs [17]. These reports, not only suggest the usefulness of the study of the blink reflex as a subtle neurophysiological outcome on central brainstem changes that occur in migraine patients, but also, the potential modelling of these changes with the infusion of GTN.

## Methods

### Aim

In rodents, the blink reflex can be elicited by electrical stimulation of the supraorbital nerve or by electrical or physical stimulation of the corneal surface and/or palpebral areas. In this study we aimed to investigate the blink reflex in rodents during GTN infusion, an established model of migraine, and to determine whether such reflexes may prove useful as the basis for a novel non-invasive animal model to evaluate potential anti-migraine therapeutic agents.

All experimental procedures were carried out at King’s College London in accordance with UK Home Office guidelines for the use of animals in experimental procedures and with approval of the local ethical committee at King’s College London.

### Animals

Male Sprague-Dawley rats (300–375 g) were housed in groups of three under controlled conditions on a 12 h light-dark cycle, with ad libitum access to food and water. A total of 31 animals were used in the study. Of these animals 23 provided data that was considered suitable for entry into the study. Data from the remaining 8 animals was not entered. In all cases where data was not entered into the study the reason was that baseline recordings were not considered to be sufficiently stable and reproducible measurements of EMG could not be obtained.

In preparation for recording, animals were brought from the holding facility to the laboratory on the day of experiment, weighed and anaesthetised with urethane (1.25 g/kg i.p). This anaesthetic was chosen as it provides a stable level of anaesthesia for many hours after initial induction with no need for supplemental dosing. The trachea, and left femoral artery and vein were cannulated to facilitate, respectively, maintenance of clear airway; recording of systemic arterial blood pressure; and intravenous administration of pharmacological agents. Animals were placed on a homeothermic heating blanket and the core temperature monitored and kept at 37 ±1^o^C by means of feedback control system (Harvard Apparatus, UK). No securing framework or support was necessary to maintain the position of the animal during the experiment. Animals were allowed to ventilate spontaneously with room air.

### Electromyographic (EMG) Recordings

Electrodes for recording electromyographic (EMG) activity consisted of teflon-coated silver wires melted to form a ball at the tip. The fur over the left upper eyelid was gently clipped close to the skin using fine scissors and with the aid of a surgical microscope and the silver ball was arranged with a micromanipulator such that its surface was pressed lightly onto the surface of the skin of the eyelid, over the orbicularis oculi (OO) muscle. Good electrical contact was facilitated by applying a small amount of electrode gel (SignaGel, Parker Laboratories, USA) between the skin and the silver ball. An indifferent electrode was placed in a similar fashion approximately 8–10 mm away, on the skin near the midline of the skull.

### Air puff evoked blink reflex

**Fig. 1:**
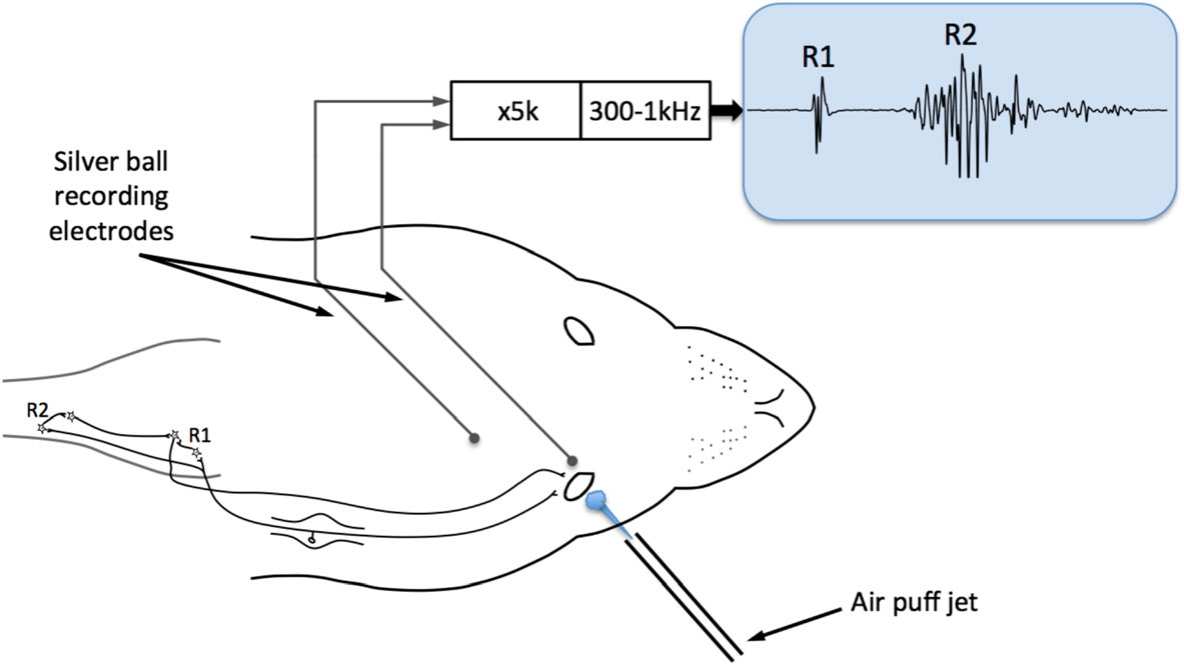
Schematic showing position of air puffer and recording electrodes with a simplified representation of the neural pathway involved in R1 and R2 components of the blink reflex. Afferent signals from sensory fibres innervating the cornea and the skin at the palpebral margin travel via the supraorbital nerve (1st division of trigeminal nerve) and trigeminal ganglion to the brainstem. Efferent signals generating the blink response travel in fibres of the facial nerve (cranial VII) from its motor nucleus to the orbicularis oculi muscles

**Fig. 2:**
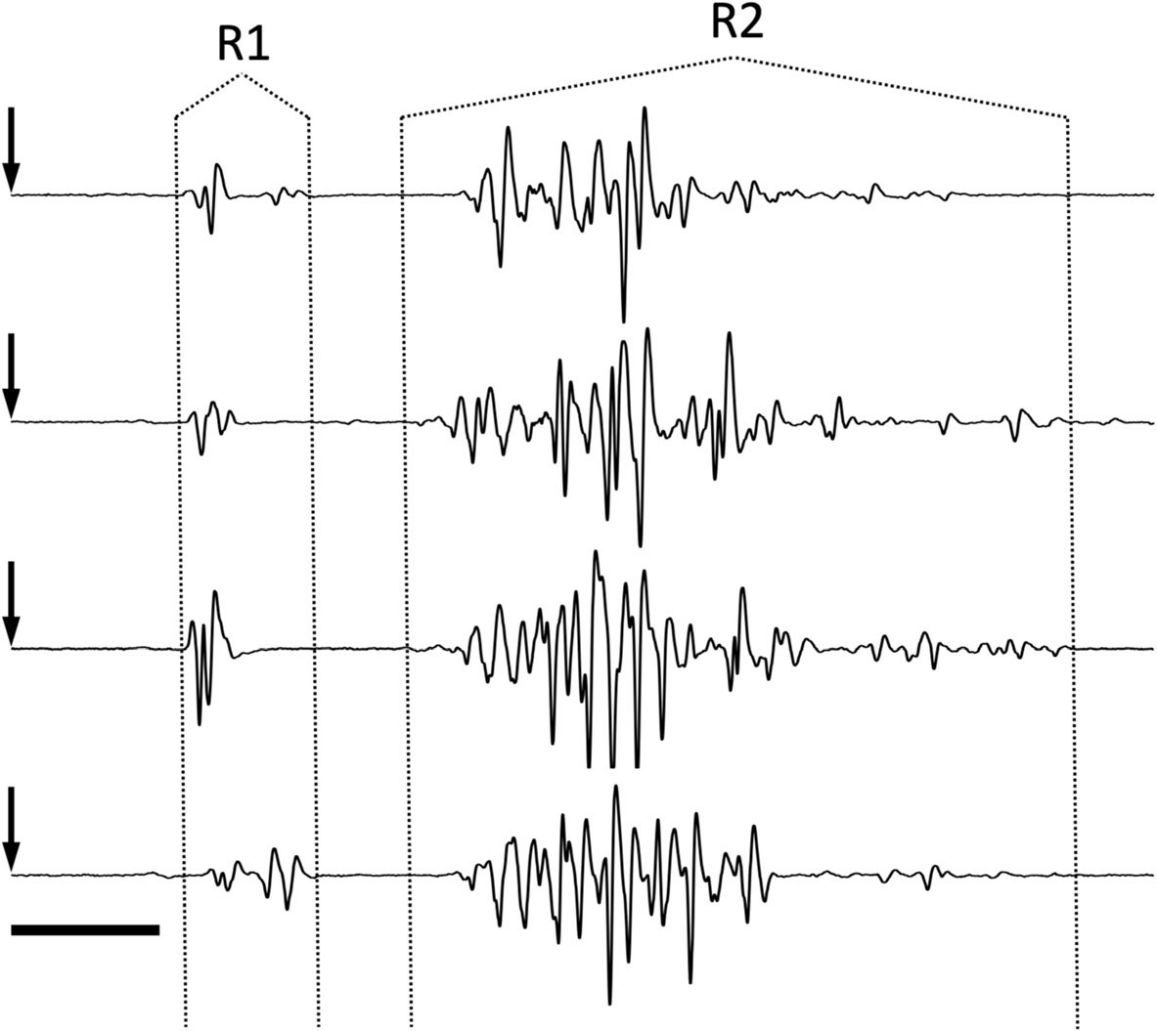
EMG traces showing (top to bottom) consecutive responses to single brief air puff directed at the centre of the cornea/palpebral margin at intervals of 15 min. The R1 and R2 components of the blink reflex are clearly demarcated and the variability in both components is illustrated in this example. *Arrows* indicate time of air puff delivery; horizontal scale bar = 10 ms

**Fig. 3:**
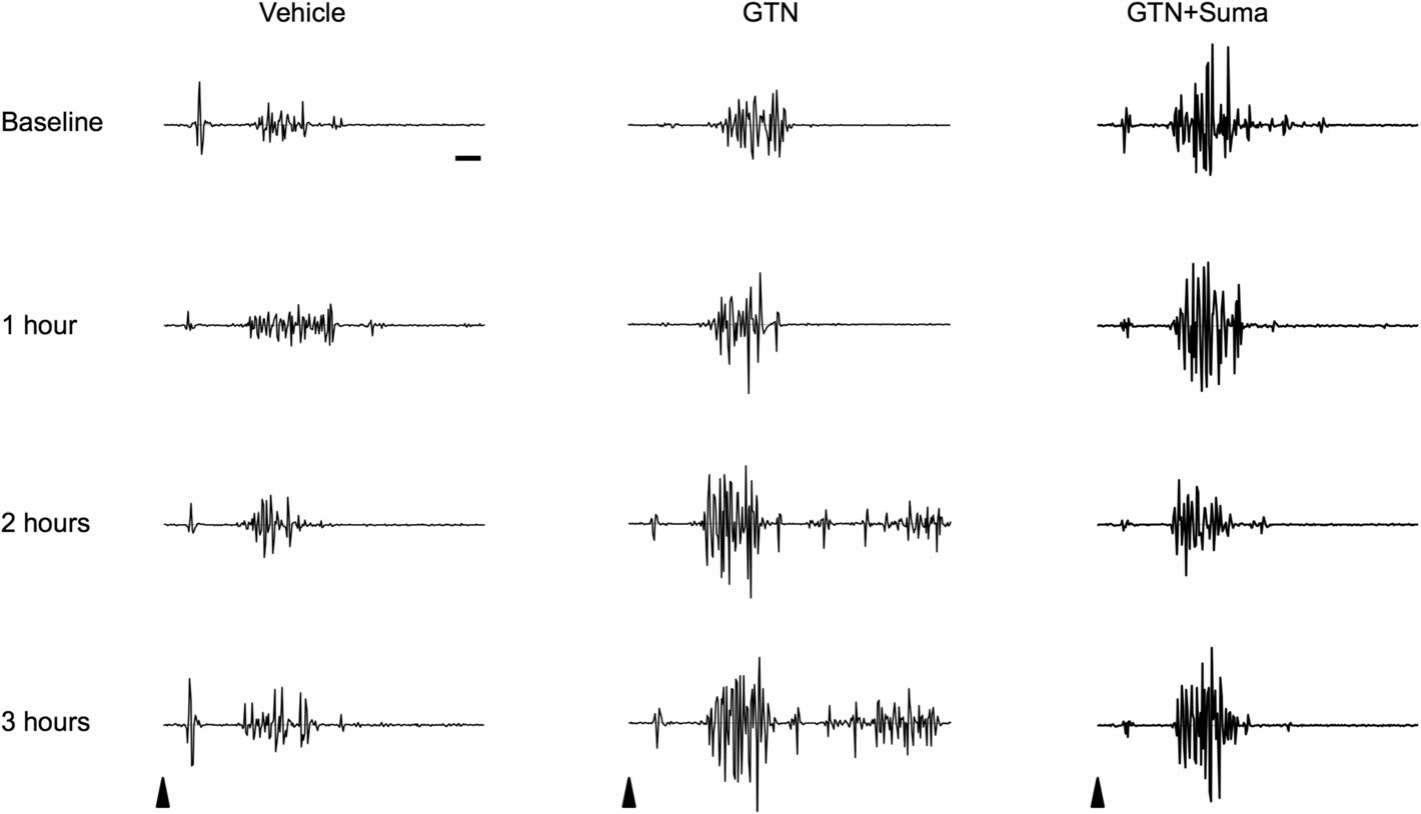
Raw EMG traces showing activity in the ipsilateral suprapalpebral orbicularis oculi muscle evoked by air puffs. Each trace represents a single sweep recorded during each of the four 1 h epochs - baseline up to 3 h after the start of infusion. Following GTN infusion, there is a progressive increase in the intensity of the reflex EMG response. Co-infusion of sumatriptan with GTN completely inhibits this effect. By comparison, the EMG remains stable after saline infusion. *Arrows* indicate timing of stimulus; horizontal scale bar = 10 ms

Reflex blinks were evoked by brief air puffs directed onto the surface of the cornea and the upper palpebral margin using a custom-built air jet apparatus engineered in our laboratory. The jet nozzle was positioned 5–6 mm from the surface of the cornea (Fig. 1) and a single puff stimulus was delivered once every 15 min to minimise sensitisation of the cornea due to repeat stimulation. Where necessary, the cornea was kept moist throughout the experiment by the occasional application of artificial tear fluid (Scope Ophthalmics, UK). Any excess moisture that accumulated over the corneal surface was gently wicked away prior to the application of a puff stimulus. The EMG of the orbicularis oculi muscle (OO-EMG) was amplified (x5k) and filtered (bandpass 300–1000Hz) using Neurolog equipment (Digitimer, UK). Examples of these EMG traces can be seen in Figs. 2 and 3.

The response to each stimulus was monitored on a storage oscilloscope (Gould, UK) and recorded with a computer running Spike 2 software (CED, Cambridge, UK). After 1 h of baseline recording, vehicle (0.9 % saline), GTN (150 μg/kg) or GTN plus sumatriptan (300 μg/kg) were infused over a period of 30 min. In all cases the infusion volume was 2 ml/kg. Following the start of drugs infusion, stimulation at the rate of 4 puffs per hour was continued for 3 h.

Considerable variation between animals was observed in the magnitude of evoked OO-EMG. For the purposes of statistical comparison, responses were rectified off-line and averaged in 1 h epochs. The magnitude (area under curve of mean rectified EMG) of the combined R1 + R2 OO-EMG response at each hour post-infusion was compared to baseline for each animal.

### Pharmacological agents

Glyceryl trinitrate solution for injection (Nitronal, 1 mg/ml; Merck, UK) was obtained from Guy’s Hospital Pharmacy and diluted appropriately in 0.9 % saline to give a dose of 150 μg/kg in a volume of 2 ml/kg. Sumatriptan succinate (Imitrex, 12 mg/ml, GSK, UK) was obtained from Guy’s Hospital Pharmacy and diluted appropriately in 0.9 % saline for a dose of 300 μg/kg in a volume of 2 ml/kg.

### Statistical analysis

The effect of treatment and time was examined by using two-way analysis of variance (ANOVA) to compare all treatment groups and all time points. More targeted tests were performed using one-way ANOVA with Bonferroni’s *post hoc* test for multiple comparisons. *P* values of less than 0.05 were considered to be significant.

## Results

All values for OO-EMG are expressed in arbitrary units (V.sec) or as percentage changes from baseline for the treatment groups at different time points. Mean values are accompanied by standard error of the mean. Two-way ANOVA of all raw data from each time point in the three treatment groups indicated a highly significant effect of treatment (*P* < 0.05; *F*_2,80_ = 1.98). Representative examples of traces from each time point in all treatment groups are shown in Fig. 3.

1. Infusion of vehicle does not alter the magnitude of the EMG

Vehicle (0.9 % saline) was administered in 7 rats. As seen in the example in Fig. 2, there were no significant changes in the magnitude of OO-EMG from baseline (range 0.0079–0.0509 V.sec) at any time point up to 3 h after the beginning of infusion (range at 3 h 0.0053–0.0588 V.sec; *P* = 0.62, *F*_3,18_ = 0.61, one-way ANOVA). The group percentage changes at these time points are summarised in Table 1.

**Table 1:** Summary of changes in magnitude of OO-EMG response in different treatment groups at different time points

Treatment	Percent change from baseline post-treatment		
	1 h	2 h	3 h
Saline (7)	-5 ± 9	+20 ± 12	-2 ± 9
GTN (10)	+7 ± 14	+42 ± 17	+55 ± 14^a^
Sumatriptan + GTN (6)	+11 ± 21	-5 ± 20	-5 ± 14

^a^Denotes significant difference with respect to GTN

2. Infusion of GTN significantly amplifies the magnitude of the EMG

**Fig. 4:**
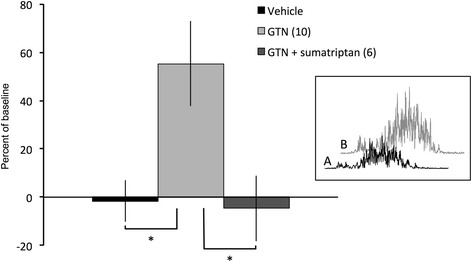
Histogram comparing the effects of vehicle, GTN and GTN plus sumatriptan on the magnitude of the evoked OO-EMG (combined R1 + R2 components) at 3 h post-infusion. Data were compared by one-way analysis of variance, with Bonferroni’s *post-hoc* test for multiple pairwise comparisons (* *P* < 0.05). Bracketed values, *n* for each group. Inset: Examples of rectified OO-EMG recorded in rat receiving GTN infusion at baseline (*A*) and at 3 h after infusion of GTN (*B*)

The effect of GTN was tested in 10 rats. Comparison of the raw data values indicated a significant overall effect of the treatment (*P* = 0.0034, *F*_3,27_ = 5.81, one-way ANOVA within group). Compared to baseline (range 0.0049–0.0398 V.sec) there was an increase of 42 ± 17 % in the magnitude of the OO-EMG at 2 h after the start of the 30 min infusion period (range 0.0049–0.0463 V.sec), however this did not reach significance (Table 1). At 3 h (range 0.0069–0.0469 V.sec), the increase in response magnitude was significant at 55 ± 14 % of baseline *P* < 0.05, *t*_20_ = 3.25; Table 1). At this time point, the increase in the response was significantly greater than in either the vehicle-treated group or the group in which sumatriptan was co-infused with GTN (*P* < 0.005, *F*_2,20_ = 7.86; one-way ANOVA between groups; Fig. 4).

3. Co-infusion of sumatriptan with GTN prevents the GTN-evoked increase of the magnitude of the EMG

Co-infusion of GTN with sumatriptan was performed in 6 rats. In this group there was no significant change in the magnitude of the OO-EMG from baseline (range 0.0175–0.0618 V.sec) at any time point up to 3 h post-infusion (range 0.0168–0.0398 V.sec; *P* = 0.73, *F*_3,15_ = 0.44, one-way ANOVA within group), and this was significantly different when compared to the response of GTN alone (*P* < 0.05; *F*_2,20_ = 7.86; Fig. 4).

## Discussion

Developing a non-invasive migraine animal model that can reliably and easily demonstrate alterations of trigeminal and brainstem function has been a long unmet need in the migraine research field. In this manuscript we demonstrate that the blink reflex in the rat represents a subtle and simple electrophysiological measurement to detect altered activity of the trigeminal-brainstem reflex in response to infusion of a nitric oxide donor. In our model, corneal air puff reliably evoked the blink in the anaesthetised rat and silver wire electrodes resting on the skin surface provided a completely non-invasive method of recording the reflex EMG activity from the OO muscle.

Recording of the blink reflex is relatively simple methodologically; requires a lesser degree of training for successful implementation than, for example, single unit recording; and could therefore potentially be adopted in a wider number of laboratories. Use of air puff as a stimulus in this study demonstrates that non-nociceptive blink reflex activity evoked in this way can be stable over a prolonged period and is susceptible to pharmacological manipulation by agents known to impact neurotransmission in the trigeminocervical system.

NO donors such as GTN have been shown to trigger an early onset headache and migraine attack in sufferers after a delay of hours [26, 42]. In healthy subjects, a milder early-onset headache is reported [43], which is also associated with decreased thresholds to mechanical nociceptive stimuli [41]. Olesen and Ashina [32] suggest that normal individuals may develop a migraine-like attack given exposure to sufficient environmental and chemical stimuli. Hence, the infusion of relatively higher doses of GTN in rodents has been used as an animal model of migraine [8].

A number of different techniques have been used in the animal GTN infusion model for the detailed exploration of the systems involved, and different end points have been considered. By means of immunohistochemistry, expression of Fos, the protein product of the early gene *c-fos*, has been widely used to study activation of the systems involved. In rodents, systemic NO donors, have been shown to induce Fos expression in the trigeminocervical complex , brainstem and hypothalamus [37, 39]. A limitation of the Fos marker however, is that its expression in any tissue, after any stimulus peaks at 2–4 h [45] and incorrectly its presence in the TCC at the 4 h time point has been thought to reflect the delayed onset of a migraine attack in humans. Although immunohistochemistry is employed by many laboratories, it has significant drawbacks. It is a time-consuming and labour-intensive technique; the timing of observations must be carefully controlled; and no more than a single trial is possible in each animal as sacrifice is necessary to obtain tissue for analysis. Our dose of GTN is considerably smaller than the dose chosen in experiments that administered GTN as an i.p. bolus injection [38, 39] but is 2.5 fold higher than the dose selected by Offenhauser et al. [31]in a study that failed to induce Fos expression. We chose the intravenous route of administration in order to deliver GTN more effectively into the circulation and used a dose of 150 μg/kg in an attempt to maximise the chance of observing effects on blink reflex recordings, whilst minimising cardiovascular effects.

Behavioural studies with GTN in animal models have demonstrated a delayed occurrence of allodynia and hyperalgesia in freely-moving animals, and photophobia and facial expressions of pain have also been studied as endpoints [11, 23]. However, recording of behavioural endpoints can be highly subjective and many animals may be required in order to see statistically significant change. With our model we believe that there is less subjectivity in the measurement of EMG responses and, importantly, we were able to see highly significant results with relatively small numbers of animals in each group. Therefore, our model may also contribute, albeit in a modest way, to the reduction of animal use.

Electrophysiological techniques have been infrequently applied in conjunction with GTN administration in laboratory models. In a preliminary report, Akerman and Goadsby [3] demonstrated that GTN infusion induced a delayed increase of spontaneous and evoked firing of second order neurons in the trigeminocervical complex, by means of single neuron electrophysiology. The techniques involved however, are highly invasive and require a greater level of expertise than immunohistochemistry [6]. Additionally, as neuronal sensitisation usually occurs after 2–4 h of GTN infusion, such an approach requires a greater effort in keeping stable single neuron recordings for multiple hours and the data yield is correspondingly low. In our model, we demonstrated that the blink reflex EMG can be a reliable endpoint to record in the GTN infusion model over several hours with no special measures required to maintain mechanical stability. We have clearly shown that GTN infusion significantly increased the amplitude of the blink reflex induced in response to corneal air puff over time, likely reflecting sensitisation of trigeminal centres in the brainstem. Interestingly, by measuring blink reflexes in human subjects it has been shown that GTN excites brainstem structures involved in migraine in humans [17, 30]. Additionally, in a randomised double-blind study measuring the nociceptive withdrawal reflex, NO donors were shown to sensitise brainstem and trigeminal systems in migraine patients [34]. Hence, in our model the use of the blink reflex enhancement as an endpoint following GTN infusion, represents a valid surrogate for human migraine pathophysiology. It is worth investigating in the future if other migraine provoking agents, such as CGRP, could also influence the amplitude of the blink reflex.

There are undoubtedly some limitations to this study. We found that the R1 component was not always as prominent as the R2 component and often showed considerable variation over the course of multiple trials. Accordingly, the combined rectified activity of both R1 and R2 was considered appropriate for analysis, as the R2 component is always much larger and this helped to minimise natural variability. Notwithstanding the variability, we did not observe any consistent changes over time (i.e. diminution of the response) that would suggest habituation was occurring. Also, whilst other studies have mainly focussed on the nociceptive blink reflex, in this study we examined only the response to air puff, which we consider to be a non-nociceptive stimulus. Overall therefore, in this study it was difficult to explore effects on each component of the reflex. The use of a nociceptive stimulus, like cutaneous electrical stimulation for example, that may permit more accurate measurement of the R1 and R2 latencies, would add additional information that could be recorded in our model, and we do plan to pursue this in the future.

The experiments described here were performed in anaesthetised rats as the blink reflex is preserved under anaesthesia in humans and animals. However, the potential also exists for this model to be adapted for non-anaesthetised animals. This would require subcutaneous, chronically implanted and, ideally, telemetrically connected electrodes; however, possible compromise of the reflex by anaesthetic agents could thereby be avoided and this is worth investigating in the future. Additionally, we plan to investigate whether the blink reflex can be reliably activated by other non-nociceptive vectors, such as fluid droplets applied to the corneal surface, or photic stimuli.

Headache induced by a nitric oxide donor in humans responds to sumatriptan and thus can be used as a model for the development of migraine drugs [25]. In the GTN animal model, sumatriptan has been shown to alleviate behavioural changes [10] and reduce Fos expression in the trigeminocervical complex [35]. In our study we demonstrated that sumatriptan inhibits the potentiation of reflex blinks following GTN infusion. Of interest, zolmitriptan, was found to reverse blink reflex changes observed during a migraine attack [15], suggesting that our animal model of blink reflex could be used to explore the efficacy of newer migraine drugs.

## Conclusions

We have demonstrated that a simple method using silver wire electrodes resting on the skin surface provides a reliable and completely non-invasive method of recording EMG activity from the OO muscle in response to activation of the blink reflex. Recordings of the blink reflex can provide an insight into the state of activation of trigeminal and brainstem structures involved in migraine and in our study GTN infusion significantly potentiated the intensity of air puff-evoked reflex blinks, an effect that was blocked by sumatriptan. Our preparation may prove a useful, novel and, importantly, translational electrophysiological model for evaluation of candidate anti-migraine compounds in the laboratory.
